# Variables Most Strongly Associated with Motor- and Health-Related Physical Fitness and Motor Skills in Five- to Eight-Year-Old Children: The BC-It and Examin Youth SA Studies †

**DOI:** 10.3390/children13050605

**Published:** 2026-04-27

**Authors:** Makama Andries Monyeki, Anita Elizabeth Pienaar, Carli Gericke, Barry Gerber

**Affiliations:** Physical Activity, Sport and Recreation (PhASRec), Faculty of Health Science, Potchefstroom Campus, North-West University, Potchefstroom 2531, South Africa; andries.monyeki@nwu.ac.za (M.A.M.); anita.pienaar@nwu.ac.za (A.E.P.); gerickecarli@gmail.com (C.G.)

**Keywords:** body composition, children, motor- and health-related physical fitness, motor skills, physical activity, socio-economic status

## Abstract

**Highlights:**

**What are the main findings?**

- Body fat percentage, moderate-to-vigorous physical activity (MVPA), and socio-economic factors (parental education, income, and employment) were the largest associated factors of health-related physical fitness (HRPF), explaining moderate to large variances (12–26%).
- MVPA contributed substantially (18.2%) to the explained variance of HRPF, with higher contributions towards aerobic fitness than strength.
- Explained variances in motor-related physical fitness (MRPF) and motor skills (MS) were modest and differed compared to those in HRPF, with sex, body composition, and school quintile status emerging as key contributors.

**What are the implications of the main findings?**

- Interventions aiming to improve children’s physical development should prioritise increasing MVPA and promoting healthy body composition, while also considering socio-economic disparities.
- A holistic, multi-level approach involving schools, families, and communities is essential to support motor and fitness development, particularly in low-resource settings and among less active groups, such as girls.

**Abstract:**

**Background:** Physical activity (PA), physical fitness (PF), and motor skills (MS) play crucial roles in overall health and well-being, particularly in early childhood, when habits that affect future health are formed. **Methods:** This study, involving 299 children (150 boys, 149 girls, mean age 6.9 ± 0.96 years), explored the variance explained by external factors such as socioeconomic status (SES), body composition (BC), sex, and geographical location on motor-related physical fitness (MRPF) and health-related physical fitness (HRPF) in children. Using a variety of assessments, including demographics, anthropometric data, BIA, ActiGraphs, the 20 m shuttle run, 10 and 20 m speed tests, and test items from the Körperkoordinations test für Kinder (KTK) and the TGMD-2, a multiple stepwise regression analysis using SPSS (v 28.0) identified the associated factors. **Results:** The variables tested show modest explained variance for HRPF, MRPF, and MS, with the largest cumulative explained variance of 26.4%. The explained variances for MRPF and MS were lower (medium to small) than the significant, medium-to-large, explained variances for HRPF. Body fat percentage (BF%), moderate-to-vigorous physical activity (MVPA), parental education and income, and BMI emerged as substantial contributors to HRPF, explaining 12.1% to 26.4% of the variance. Sex, BF%, and quintile status were the most influential associated factors for MRPF, and for MS, BMI and sex emerged as the strongest contributors. **Conclusions**: These findings underscore the importance of holistic approaches that consider individual factors, such as MVPA, body composition (BC), PA levels, sex, and broader social and economic contexts, to promote children’s well-being. The study emphasises the need for comprehensive strategies to address the multifaceted associations with children’s physical development.

## 1. Introduction

Health (HRPF), motor-related physical fitness (MRPF), physical activity (PA), and motor skills (MS) are considered strong influences on healthy behaviour and the future health of young children [1,2,3]. However, over the past few decades, there has been a global decline in physical fitness (PF), PA, and MS [3,4,5], and it remains unclear which factors contribute most to these declines. In this regard, the developmental model of Stodden and colleagues confirms the reciprocal relationship between MS and PA [6], in which they reported four correlates of MS, including perceived MS, HRPF, PA, and obesity (OB), all of which are regarded as health outcomes in children.

MS is defined as the degree of skilled performance in a wide range of motor tasks and the movement coordination and control underlying a particular motor outcome [6]. It is emphasised as a key role player in the development of HRPF and increased PA throughout childhood [6,7]. MS is also reported to promote positive trajectories of fitness and PA behaviours across childhood, adolescence, and into adulthood [6,8,9,10,11,12,13,14,15]. MS is also directly associated with HRPF from childhood to adolescence, as it reflects the ability to perform goal-directed movements that involve large muscle groups or the whole body [12,13]. HRPF is characterised by a variety of factors [body weight status, cardiorespiratory fitness (CRF), musculoskeletal fitness, muscular strength, endurance, and flexibility] and is reported to be associated with various health markers and outcomes in youth [16,17]. HRPF is found to be lower in children with poor MS in all income groups [18,19]. Furthermore, there is strong evidence of an inverse association between MS and body weight status, and positive associations have been reported between CRF and musculoskeletal fitness in children and adolescents [1,20].

PA is described as any body movement produced by skeletal muscles that results in energy expenditure [21]. Researchers found that PA at age six was positively associated with moderate-to-vigorous physical activity (MVPA) and MS by age nine, concluding that PA in early childhood is essential, as it is linked to higher levels of objectively measured PA in adolescence [22]. However, understanding the role of MS in PA and overall health promotion throughout life is complex [6,23]. It has been argued that children with underdeveloped MS have fewer opportunities to engage in PA, including sports, and later in life, they lack the necessary skills to be physically active [6,23].

Against this background of declining trends in PF, PA, and MS, and to promote optimal health and active lifestyles among children, a better understanding of the factors that may promote MRPF, HRPF, and MS is essential. These include factors such as SES (i.e., educational level, income of parents, employment status), BC [i.e., underweight (UW), overweight (OW), obese (OB)], sex, and geographical location (school quintile status). The negative influence of unhealthy BC, such as OW, is reported in various studies on the unhealthy behaviour of children aged six to 13 years old [24,25,26,27,28,29,30,31,32]. All children, even preschool children, who are OW/OB tend to have lower levels of PF and MS regardless of geographic area [20,26,33,34,35,36,37,38,39,40]. In addition, there is growing evidence that early markers of metabolic disease risk emerge in childhood and are strongly associated with OB [41,42,43]. At the other extreme, UW also negatively influences some measures of PF [44]. Furthermore, poor nutritional status (stunting in children, whether OW or not) is also thought to have a negative impact on PA, resulting in decreased PF [45].

The effects of socio-economic inequalities and unique environmental influences on PF and MS are also reported [46]. Parental education and the stability of parental employment are reported as possible significant influences on MRPF, HRPF, and MS during early childhood development [47,48]. Consequently, based on a fundamental premise about the relationship between MS and SES, children from lower-income families are more prone to experience deficits in their motor development, MRPF and HRPF [49,50,51]. In this regard, associations between insufficient financial resources and a lower level of MS were evident in a study by Peric et al. [52], in which MS correlated highly with unemployment rate, potentially linked to growing up in poorer regions. In this regard, performance in the standing long-jump test showed that higher levels of SES in early childhood can predict greater lower-limb explosive strength in early adolescence [48,53,54]. Lower limb muscular endurance was also affected by parent–child leisure activities and early childhood screen time [48]. Contradictory findings are also reported on such influences, i.e., positive associations are found between MS and SES in well-developed countries.

The geographical location (represented as school quintile status in South Africa), or the built and natural environment, is also reported to independently influence the PA behaviours of children [55,56,57]. Access to formal PA facilities differs across urban and rural contexts and schools, and researchers found that children aged 10 to 11 years residing in urban environments spent up to 14 min more per day in sedentary activities than children from rural settlements [58]. This difference was more significant during weekdays, at 16 min. The lower levels of sedentary time among rural children were offset by higher levels of light activity (a 13 min difference). This finding has been attributed to adopting urban lifestyles, introducing technology-related devices, and shifting from active transportation, particularly among children from rural areas [31,59].

Research findings also indicate significant sex differences in the movement activities of children aged 7 to 14, where girls’ PA and fitness are influenced by good locomotor skills, whereas in boys, a combination of well-developed locomotor and manipulative MS is influential [28]. Boys also engage in significantly longer MVPA than girls, who engage more in lighter activities [60,61]. These differences extend to fine and gross MS, which may be influenced by sociocultural factors such as toy choices and differences in PA engagement between the sexes at both the prepubertal and pubertal phases of development [62,63].

This background reveals gaps in understanding the magnitude of the above influences, while research on the interplay among these factors among children is sparse, especially in developing or middle-income countries such as South Africa [46,64]. Therefore, this study aimed to determine if factors related to SES (parental education, employment and income), BC, sex, and geographical location (school quintile status) have significant associations with the MRPF, HRPF and MS of five- to eight-year-old South African children, and if so, which will emerge with the strongest associations. Results from this study would provide a better understanding of the factors that may promote MRPF, HRPF, and MS in children, which, in turn, would help promote optimal health and active lifestyles among children, especially in the current age of global declines in PF, PA, and MS.

## 2. Materials and Methods

### 2.1. Research Design

This research adhered to the principles outlined in the Declaration of Helsinki. The ExAMIN Youth Study (Exercise, Arterial Modulation and Nutrition in Youth South Africa) is an analytical, multidisciplinary, observational cohort study designed to investigate the interplay between body composition, nutrition, physical activity, and biomarkers related to psychosocial stress, cardiovascular function, and salivary biomarkers. This study was retrospectively registered on ClinicalTrials.gov (accessed on 19 April 2026, https://clinicaltrials.gov/study/NCT04056377) (NCT04056377) [65]. The BC–IT study examined the relationships between more complex markers (using a stable isotope method and Bioelectrical Impedance Analysis (BIA)) and more indirect measures of body composition (using anthropometric variables), and the BC–IT study [66,67] is not a clinical trial study (Figures S1 and S2). It also examined objective and subjective measures of physical activity, physical fitness, and other health-related determinants among South African children aged 5–8 years.

Approximately 1000 children aged 5–8 years enrolled in 10 public primary schools (i.e., for Clinical Trial study) within the Kenneth Kaunda District (Potchefstroom and Klerksdorp), North-West Province, South Africa, were included. This sub-study employed a cross-sectional design (based criteria methods for determining body composition mainly in five primary schools—2018/2019), utilising available data from the BC-IT and ExAMIN Youth SA studies. The analysis focused on the interrelations and contributions of demographic and biological characteristics and MVPA as possible enablers of MRPF, HRPF and MS in children’s early health.

### 2.2. Research Group

This study utilises a subsample from the larger ExAMIN Youth and the Body Composition (BC) by Isotope Techniques (BC–IT) studies, including 299 children (150 boys and 149 girls) aged between five and eight years with complete data on BC using an isotope technique [the Body Composition–Isotope Technique (BC–IT) study; 2018/2019]. As such, 299 children from the BC–IT study had complete data on the Deuterium Dilution Method (DDM), bioelectrical impedance analysis (BIA), ActiGraph, and MRPF, HRPF, and MS measurements, and were included in the study. As described in the larger BC–IT study [66], the variables in this study are drawn from the five primary schools randomly selected from 26 schools in the JB Marks municipal area (Potchefstroom municipality) in the Kenneth Kaunda district of the North-West Province, South Africa. The ExAMIN Youth Study expanded its sample to 1000 participants by including children from additional schools both within and outside the Potchefstroom municipality in the Kenneth Kaunda district. These schools represented different school quintiles (3 [low]–5 [high]). The Generalised Linear Model for Analysis of Variance was used to calculate the statistical power for the appropriate sample size, with a power of 0.80 and a level of 0.05 at a CI of 95%. Every third child on each class list was selected for the study. Still, only those with signed parental informed consent forms who personally agreed to participate were finally included. The mean age of this subsample was 6.83 (±0.96) years, including participants in the following age groups: 5 years (n = 27), 6 years (n = 86), 7 years (n = 95), and 8 years (n = 91).

### 2.3. Ethical Approval

The Health Research Ethics Committee of the Faculty of Health Science at the North-West University, Potchefstroom, SA, granted permission for the observational cohort/follow-up study, the BC-IT study (NWU-00025-17-S1), the ExAMIN Youth SA (NWU-00091-16-A1), and this sub-study (NWU-00457-20-A1). The Department of Basic Education, school principals, parents, and children approved it. The parents/legal guardians had to provide written consent, and participants had to provide verbal or written assent to participate in the study. The permission form addressed to the parents/legal guardians explained what would be expected of the participants and outlined the risks involved. On the day of data collection, the steps and procedures were briefly explained to the participants. Each participant received a participant number and data collection sheets.

### 2.4. Measuring Instruments

#### 2.4.1. General Health and Demographics Questionnaire

Socio-demographic information, including personal information and information on family (i.e., educational level, parents’ income, employment status, type of dwelling, and marital status), lifestyle behaviours, and health, was collected using a standard general health questionnaire. Parents or legal guardians assisted with completing the demographic questions. When responding to the SES questions, the parents had to choose from several options: What level of education have you completed? (none, primary school, high school, adult basic education training, or tertiary institution). Which of the following applies to your current employment situation? (unemployed, employed, or self-employed), and what the total household income is per month.

Socio-economic status (SES) in this study was obtained using parental education, employment status, and household income, as these are widely used and practical proxy indicators in large-scale field-based research. These measures capture key dimensions of SES, including parental income, household resources, and employment status. However, it is acknowledged that SES is a multidimensional construct, and the use of a limited set of indicators may not fully reflect broader contextual factors such as wealth, neighbourhood environment, or access to resources, which should be considered when interpreting the findings.

#### 2.4.2. Anthropometric Measurements

The standard procedure prescribed by the International Society for the Advancement of Kinanthropometry (ISAK) was used to determine participants’ anthropometric measures of height (cm) and weight (kg) [68]. Level 2 anthropometrists took these measurements. Separate rooms were used for the boys and girls to ensure privacy. A Seca 213 stadiometer (Holtain Limited, Crosswell, Crymych, UK) was used to measure height to the nearest 0.1 cm, with participants barefoot and standing upright with their heads in the Frankfort plane, after inhaling. Weight was measured to the nearest 0.1 kg with a Seca 813 digital scale (Beurer Ps07 Electronic Scale, Ulm, Germany) while participants wore minimal clothing and no shoes. Two measurements were taken for each indicated variable, and the average of the two was used for the analyses. The BMI was calculated using weight and height measurements (weight/height^2^), whereafter, the BMI z-score was calculated relative to WHO reference data [69].

#### 2.4.3. Physical and Motor Fitness and Motor Skills

This testing protocol included tests from different test batteries to assess HRPF, MRPF and MS [26,70,71,72]. The use of multiple assessment tools (KTK, TGMD-2, and fitness tests) was intentional to provide a comprehensive evaluation of the distinct but related constructs of MS, MRPF, and HRPF. Each instrument captures different dimensions of children’s physical development—process- and product-oriented motor competence (TGMD-2), coordination and balance (KTK), and physiological fitness components (fitness tests). This combined approach aligns with the multidimensional nature of physical development and allows for a more holistic assessment of the variables under study.

Health-related physical fitness (HRPF)

Three HRPF characteristics were tested, including the 20 m (m) shuttle run test (20 m SRT), predicted V^·^O_2_max in millilitres of oxygen per minute per kilogram of body weight (mL/kg/min), and leg strength. The 20 m SRT is a valid and recognised endurance test that shows reliability in children aged six to 16 (r = 0.89). The test involves running back and forth across a 20 m distance [70]. The starting speed is 8.0 km/h, with a 0.5 km/h rise every minute, paced by beeps on a stereo. A final score is taken when a participant drops out because of exhaustion or cannot cross the 20 m line at the point of the beep for two consecutive 20 m lengths. An indirect aerobic capacity score (V^·^O_2_max) was calculated using the FitnessGram equation, including field test scores, age, sex, and BMI [70]. The following equation was used to convert the attained beep levels to predict aerobic capacity: V^·^O_2_max 45.619 + (0.353 × Pacer laps) − (1.121 × age).

Motor-related physical fitness (MRPF)

MRPF was tested using running speed and agility measurements. A 10 m and 20 m speed test determined running speed using electronic timing gates from Smartspeed, Fusion Sports, Summer Park, Queensland, Australia, which have a reliability of 0.9 in children aged 6 to 11 years. After an acoustic signal, the participant starts the 20 m run from a standing position. The time, in seconds, to complete the 10 m and 20 m sprint tests was recorded as a quantitative measure of running speed, with the best of two trials scored. Agility was also assessed quantitatively using a two-legged jumping sideward test from the Körperkoordinationstest für Kinder (KTK) [71], in which the number of successful jumps was scored over 15 s.

Motor skills (MS)

Process (quality) and product (quantity) performance in MS were evaluated through running, jumping, catching, kicking, and balancing, selected to provide a comprehensive overview of participants’ MS abilities. Because of time constraints, only four tests representing two locomotor skills (running and jumping) and two object-control skills (catching and kicking) were used from the Test of Gross Motor Development (TGMD-2) protocol [72] to assess MS qualitatively (process). It is not uncommon for researchers to select only a few of these skills for their studies. Jumping represented a qualitative measure of leg strength in HRPF, and running represented a qualitative measure of running speed in MRPF. The catching and kicking skills represented qualitative measures of motor skills for object control. These four skills are also the most common, most often chosen, and most relevant to typical South African sports and game activities. Following the TGMD-2 protocol, the skills of running, jumping, catching and kicking were demonstrated. Then, two attempts were allowed and scored according to specific sub-criteria (0 = no mastery; 1 = mastery), after which the scores were summed. Standard sub-criteria of the TGMD-2 protocol were used to assess running (4 sub-criteria), jumping (4 sub-criteria), catching (3 sub-criteria), and kicking (4 sub-criteria), as described elsewhere [71]. Balancing was scored qualitatively out of 3 (1 = initial phase, 2 = elementary phase, 3 = mature phase) using the Kinderkinetics protocol described elsewhere [26].

A product assessment evaluates a movement’s outcome, typically identified as a quantitative score (e.g., speed, distance, or number of successful attempts). Product-oriented evaluations of this study protocol included running speed in seconds, scored as the best of two trials, for the 10 m and 20 m run tests. Catching and kicking accuracy were scored out of five attempts using the TGMD-2 protocol (distances between the tester and the participant in the catching skills and the distance to the kicking target of 1.5 cm wide). The distance jumped in the horizontal jumping test (SBJ) of the TGMD-2 was used as a quantitative measure for jumping. Two trials were allowed, and the best trial was recorded in centimetres. This test was performed on a non-slippery mat explicitly designed for horizontal jumping. Two of the four KTK test items [71] were used to obtain quantitative scores of balance and agility. Balancing was tested by walking backwards along a balance beam of decreasing width (6.0 cm, 4.5 cm, and 3.0 cm), and the number of successful steps was counted. Agility (MRPF) was assessed by the number of successful sideward two-legged jumps performed in 15 s [73].

All assessments were conducted by trained researchers in Kinderkinetics, with participants rotating between stations. The same evaluators were used for each test item to reduce tester variability and improve the reliability of the results. The 20 m shuttle run was done after all measures were taken to minimise fatigue effects on the various tests. On the day of testing, all the participants were transported by bus from their school to the PhASRec laboratory at the university, tested, and then returned to the school before the end of the school day.

#### 2.4.4. Physical Activity Using ActiGraph Accelerometers

PA and sedentary behaviour were assessed using the ActiGraph GT3X (model 7164; Fort Walton Beach, FL, USA), a solid-state triaxial accelerometer validated for use in children. According to the manufacturer’s instructions, each participant was fitted with the ActiGraph device, which they had to wear around the waist just over the anterior superior iliac spine. Participants had to wear it for at least 10 h/day for seven consecutive days. The participants were, however, permitted to remove the accelerometers during water-based activities or during sleep if they felt uncomfortable wearing the device. Parents had to complete a daily log sheet to indicate when the ActiGraph was worn and removed. Each participant also received an instruction manual on using the accelerometer for additional guidance. The ActiLife software (Version 6.13.3, Pensacola, FL, USA) was used to extract and analyse the data. The PA data were expressed as average daily minutes spent in sedentary behaviour (<99 counts per minute), light PA ≥ 100 counts per minute, moderate PA ≥ 2 296 counts per minute, and vigorous PA ≥ 4 012 counts per minute. Time in MVPA was calculated as the sum of moderate PA and vigorous PA. Participants who provided at least four days of valid data, including one weekend day, were included in the analysis. Valid days were those on which the accelerometer was worn for at least 600 min (10 h) daily. Consecutive zero counts for 20 min or more were considered as no-wear time.

#### 2.4.5. Body Composition by Bioelectrical Impedance Analysis (BIA)

BC was assessed using BIA (Bodystat 1500MDD, MultiScan 5000, BodyStat Ltd., Douglas, Isle of Man, British Isles), following the manufacturer’s protocol as previously published [74]. Participants were asked to refrain from exercising or playing in the sun for 8 h before the procedure. The measurement frequency was set at 50 kHz, and height, sex, and age were entered manually, while body mass was recorded automatically using 0.5 kg to adjust for clothing weight in all subjects. Participants were asked to remove jewellery and belts containing metal or metal-rimmed glasses before lying quietly and without motion on a non-metal examination table or bed on their backs, with arms flexed to the side and thighs not touching. Detection electrodes were placed at the pisiform prominence of the wrist and the anterior surface of the true ankle joint, after wiping with a moist antiseptic towel (Right side, as recommended) [66,74].

The Bodystat software uses inbuilt prediction equations to produce an output specifying total body water (TBW) in litres, FFM (kilograms), BF mass (kilograms) and %BF, as well as impedance, resistance, and reactance readings. The existing BIA equations were used to estimate TBW. Children were classified as normal (FM% 14.9% to 24.9), UW/Thinness FM% < 14.5%, OW (FM% > 30 & 34.9) and OB (FM% > 35%) in line with the cut-points of McCarthy et al. [75] and Williams et al. [76].

The inclusion of both BMI and BF% was justified because BF% was determined using Bioelectrical Impedance Analysis (BIA), whereas BMI, as a surrogate measure of body fatness found to be associated with misclassification of fatness, was derived from the height-to-weight index. BIA appears to be more suitable for assessing body fatness because of its apparent advantages and the availability of validation studies.

### 2.5. Statistical Analysis

A multiple stepwise regression analysis was used in SPSS version 28.0 to determine the relevant contribution of SES (educational level, income of parents, employment status), BC (UW or OW), sex, and geographical location (school quintile status) associated with outcome variables, MRPF, HRPF, and PA in five- to eight-year-old children. SES, BC, sex, and MVPA were included as possible covariates in the analysis. To enhance the normality and homoscedasticity of the residuals, logarithmic transformations were applied to the MRPF, HRPF, and MS dependent variables. The strength of the relationship between MRPF, HRPF, and MS in five- to eight-year-old children controlling for SES, BC, sex, MVPA, and geographical location during the stepwise regression analysis is presented as the percentage variance explained, where R^2^ ≈ 1% can be interpreted as a small effect, R^2^ ≈ 10% as a medium effect, R^2^ ≥ 25% as a large effect. For statistical significance, *p* is set at ≤ 0.05.

Multiple stepwise regression was used to determine the most relevant predictors of MRPF, HRPF, and MS from a set of interrelated predictors in a relatively large, cross-sectional dataset. This method allowed for the selection of variables that contributed most substantially to the explained variance while reducing model complexity, by retaining only variables that made a statistically significant contribution to the explained variance.

## 3. Results

Table 1 summarises participant characteristics. The mean age was 6.83 (±0.96) years, with no significant age differences between boys and girls (*p* = 0.75). No sex differences were observed for height, weight, BMI, or waist circumference (*p* > 0.05). However, girls had significantly higher body fat percentage (BF%) than boys (*p* < 0.001), while boys had higher fat-free mass (*p* < 0.001). Based on BMI classification, 11% and 8% of participants were overweight and obese, respectively. Girls showed higher rates of overweight and obesity, whereas boys were more frequently underweight (*p* < 0.05).

Table 2 presents MRPF, HRPF, and MS outcomes. Significant sex differences were observed in most variables (*p* < 0.001), except for agility and balance quality. Boys performed better in speed, HRPF measures, and object control skills (catching and kicking), while girls outperformed boys in balance quantity. No differences were found for agility or balance quality.

Table 3 presents the objectively measured PA behaviour characteristics of the group and by sex. This analysis showed significant sex differences (*p* < 0.001), with boys (n = 150) being significantly more moderately (54.18 ± 17.12) and vigorously (24.10 ± 64.73) active than girls (45.38 ± 13.35 and 19.93 ± 10.65). Boys also displayed significantly (*p* < 0.001) higher mean total MVPA (71.82 ± 25.21) and average MVPA (78.28 ± 26.89) per day, with 73% of boys meeting the recommended daily MVPA compared to only 58% of the girls (*p* < 0.002). Also, boys had significantly (*p* = 0.05) higher mean (793.73 ± 55.09 min) wear time than the girls (781.68 ± 50.95 min). No significant (*p* > 0.05) sex differences were found in sedentary and light PA behaviour.

Regression analyses for HRPF (Table 4) indicated that MVPA, BF%, and SES-related variables (education, income, and employment) were associated with HRPF outcomes, while sex and geographical location were not retained in the models. The explained variance ranged from 12.1% to 26.4% (medium to large effects). MVPA emerged as the strongest associated factor for aerobic fitness (Pacer and V^·^O_2_max), while BF% consistently contributed across all HRPF outcomes. For standing long jump, additional contributions were observed from parental education, income, and BMI.

In summary, these results underscore the significant role of MVPA, BF%, and SES in associations with parental education, income, and employment in HRPF, with distinct effects observed in SBJ, Pacer laps, and V^·^O_2_max performance. Neither sex nor geographical location was considered in any of these models.

Table 5 presents the prediction models generated for the MRPF and MS test items. The explained variance for MRPF and MS was lower than in the HRPF test items, ranging from small to medium effects for MRPF (speed and agility) and mainly small for MS (balancing and catching), except for kicking skills. In kicking skills, sex alone entered the model, explaining 9.3% of the variance, which is a medium-sized significant effect. The associated factors that entered the models also differed, mainly based on the HRPF models’ findings regarding the contribution order. Here, sex, BF%, school quintile status (thus environmental effects), BMI, and to a lesser extent MVPA, parental income, and employment status emerged as associated factors in MRPF and MS. Sex was the primary predictor in speed and agility (speed 10 m = 9.3%; speed 20 m = 10.2%). The effect of school quintile status was evident across all four MRPF tests (speed 10 m = 2.7%; speed 20 m = 2.6%; agility = 2.6%; balance quantity = 6.2%), with a combined contribution of 14.5% to overall MRPF tests were when stratified by sex. For 10 m speed test, sex, school quintile status, and BF% were included into the regression model, collectively explaining 12.7% of the variance in performance (adjusted *R*^2^ = 0.127). In the 20 m speed, sex, school quintile status, BF%, and MVPA were associated factors. These four associated factors explained 15.8% of the 20 m speed variance (final adjusted *R*^2^ = 0.161). The coefficient (*β*) indicated a significant link between sex and a 0.176 enhancement (15.848, *p* < 0.001) at 20 m speed. BF% was the sole predictor for running quality, illustrating that BF% contributed 1.7% to the running quality variance (final adjusted *R*^2^ = 0.017). MVPA, school quintile, and household were associated factors of agility, accounting for 8.9% of the variance (final adjusted *R*^2^ = 0.089). The coefficient (*β*) indicated a significant association: the three factors together increased performance by 3.092 (*β* = 3.092; 95% CI: 1.621; 4.563).

Sex, school quintile status, BMI, MVPA, and employment all emerged as factors explaining the variance of MS. BMI was the primary predictor in four of the six tests, accounting for 14.8% of the association with MS. Sex also made a big contribution, although it only emerged in the kicking quantity and quality with a combined 15.3% contribution to MS. BMI was the sole variable of eight that entered the regression model to explain some of the variance in the quality of balancing.

BMI, employment status, and school quintile were the only factors associated with the balancing total (quantity), accounting for 6.2% of the balance total for participants (final adjusted *R*^2^ = 0.062). The coefficient (*β*) indicated that a one-point increase in parental employment status was associated with a 3.409-point improvement in the balancing total. Only BMI and MVPA entered the regression model for catching (quantitative total), accounting for 6.8% of the variance. The quality of catching was associated with BMI and employment status, accounting for 7.6% of the variance. In both the quantity and quality of kicking skills models that were developed, sex emerged as the sole contributor. The adjusted *R*^2^ values showed that being male or female accounted for 1.7% of the variance in quantity and 0.089% of the variance in kicking skill quality, respectively.

## 4. Discussion

This study determines the possible influences of SES (educational level, income of parents, employment status), BMI, BF%, BC (UW and OW), sex, geographical location, and MVPA on the MRPF, HRPF, and MS in children between the ages of five and eight years. The developed regression models indicated that BF%, MVPA, parental education and income, and BMI were significantly associated factors of HRPF. In contrast, sex, school quintile status as a proxy for environmental influences, BF%, MVPA, BMI, and parental income were associated with MRPF and MS, although with less explained variance than those established for HRPF. MVPA was the most significant factor associated with HRPF, whereas sex and environmental factors (school quintile status) accounted for most of the variance established in motor-related fitness. In addition, sex and BMI were the most influential factors associated with MS. The possible predictive values of the developed models for HRPF showed medium-to-large significant contributions, whereas the explained variances in the regression models for MRPF and MS were generally lower. However, various associations were found, highlighting that the largest cumulative explained variance was only 26.6%, leaving a significant majority unexplained.

MVPA contributed to an 18.2% increase in HRPF, underscoring its significance in promoting and maintaining a healthy level of PF. However, there were differences in the contribution of MVPA to aerobic fitness and strength. Although both were relatively high, aerobic fitness was higher. These findings confirm that MVPA significantly shapes the aerobic fitness landscape among children aged five to eight years [77]. During this critical developmental period, engaging in regular MVPA may have profound positive implications for their overall health [78,79].

Strength and MVPA are two distinct components of PF, with complex, interconnected relationships. Strength and MVPA contribute to functional fitness, or the capacity to perform daily tasks efficiently. Good strength can make daily activities such as carrying school bags or climbing stairs less strenuous. In contrast, MVPA enhances overall endurance and stamina, making it easier to exercise for longer. Therefore, the relationship between strength and MVPA is one of synergy and balance. Stronger children often have greater muscle strength and endurance, which allows them to engage in more physically demanding activities [15]. They may also find it easier to perform tasks that require physical exertion, such as running, jumping, and playing sports [80]. Although physical strength can contribute to increased MVPA, it is just one factor among many that is associated with a child’s activity levels [81]. MVPA also contributed to the explained variance in speed (entered first in the prediction model, explaining 2.6% of the variance) and catching skills, although with lower predictive values than in HRPF. Children develop coordination and MS through engagement in PA, as increased PA provides more opportunities to promote neuromotor development, which in turn promotes MS development [82].

BF%, being a second associated factor in two of the HRPF test items (Pacer laps and V^·^O_2_max) and first in strength (standing broad jump), cumulatively contributed 19.2% to HRPF. An elevated BMI often corresponds to an increased proportion of body fat, which can adversely affect cardiovascular fitness, strain joints, and contribute to metabolic imbalances, potentially leading to conditions such as hypertension, type-2 diabetes, and cardiovascular disease. This highlights the impact of BC on HRPF outcomes, reinforcing the importance of maintaining a healthy BF%. BF% and MVPA also explained some of the variance in MS. BF% was associated with the quality or quantity of execution for four of the six MS, accounting for a combined 14.8% of the variance in these MS. These include running, balancing, and catching skills. Similar associations of BF% and MVPA are confirmed in other studies on children of the same age [53,83,84,85]. These findings confirm that MS is positively related to MVPA and healthy weight status in children.

SES is reported to significantly affect human physical development, including hand-eye coordination (manual dexterity), reaction time, and fine MS, which are associated with the MRPF and HRPF domains [86]. Similar results were found in this study, where educational level, parents’ income, and employment status explained some of the variance in MRPF, HRPF, and MS, with educational level showing the strongest association among SES factors. Four of the seven test items analysed in this study were associated with the parents’ employment status, with the explained variance in the MRPF and HRPF being the strongest. Employment status also contributed to the explained variance of the Pacer and V^·^O_2_max, while both educational level and familial income contributed significantly to the explained variance in strength. Aligned with our findings, the study of Wong et al. [48] revealed that having a higher family SES in early childhood predicted having more lower limb explosive strength (as evidenced by better standing long-jump performance) and lower limb muscle endurance (as evidenced by better 6 min walk test performance) in early adolescence. These researchers concluded that children from lower-SES families may be more likely to engage in sedentary activities, such as screen time, rather than recreational and physical activities, which can lead to poor PF in adolescence. It is reported that when parents overtly encourage their children to engage in PA by providing transportation, being active, watching or supervising, and/or buying equipment for it, they directly reinforce their children’s activity [87]. All these motivational factors are related to parental income. Parents from better SES can afford to support their children’s activity choices as they realise the importance of participating in sports and being active. They support children’s engagement in activities at various levels. Higher parental education may, therefore, contribute to a more nurturing and stimulating home environment, which can positively influence a child’s motor development [88,89]. In contrast, unemployment and job insecurity can lead to financial strain and stress, negatively affecting children’s mental and physical health, academic achievement, and behaviour [47]. A study on children younger than our sample (three- to five-year-olds) to identify socio-economic and familial characteristics that are associated with below-average gross motor skills [90] found that out of the socio-economic and familial factors that were investigated (ratio of family income to poverty), children living below the poverty threshold, were, contradictory to our findings, more likely to have better gross skills, indicating an inverse association between family income and gross motor skills development. Growing wealth in some cultures or communities can be linked to missing opportunities for gross motor activities, which could delay the acquisition of gross motor skills [88]. However, this advantage seems to have disappeared at older ages, at least in HRPF, according to the study by Wong et al. [48], which examined the role of early-life activities as mediators of the longitudinal association between early-life SES and HRPF among 168 adolescents with a mean age of 12.4 years.

School quintile status also emerged from the speed and balancing models. Children from low-SES backgrounds are more likely to be physically inactive [91] due to less access to outdoor play equipment such as bicycles and jumping ropes, green areas, high-quality educational programmes, and sports teams that support the development of their MS [15,48,92,93,94]. This lack of outdoor play opportunities might negatively affect their motor development [95,96].

Sex as a predictor did not contribute to HRPF. Still, it contributed to MRPF (specifically in speed) and, to a lesser extent, to some MS, while it was the only factor explaining the variance observed in kicking skills. Sex explained a small proportion of the variance in kicking accuracy (quantity: 2.2%) and, especially, kicking execution quality (9.3%). In agreement with these findings, a study by Pienaar et al. [26] on six-year-olds in the Potchefstroom and Vaalharts areas of South Africa reported an 88.6% difference in kicking competence between boys and girls (70%). Cultural and sporting preferences, as well as stereotyped play preferences, that facilitate the development of specific movement skills, can explain why boys are more proficient at kicking, especially in low-SES areas of SA. Greater exposure to street soccer and other sports that involve object manipulation, such as soccer, provides opportunities to improve kicking skills, consistent with the findings of Pienaar et al. [62,97]. However, researchers [62] have reported that during the preschool period (three- to six-years), sex differences are not absolute and can vary between individuals.

Overall, this study’s results show that BMI, BF%, MVPA, sex, SES-related factors, and the child’s school quintile status are all essential contributors to explaining variance in MRPF, HRPF, and MS in early childhood development, especially at the young age of five. The explained variances were highest for HRPF, with different combinations of associated factors explaining MRPF, HRPF, and MS in the group. These findings highlight the importance of these factors when addressing physical and motor fitness and MS development in children. It also underscores the need for future research to deepen our understanding of the complex interplay among associated factors, as the contributors analysed in this study could only explain part of the variance in MRPF, HRPF, and MS. The largest cumulative variance explained in this study was 26.6%, leaving 83% of the variation unexplained. As a result, more research is needed to identify other potential factors associated with children’s MRPF, HRPF, and MS, including sleep, nutrition, maturation, the built environment, and parental PA modelling during early and middle childhood. While the study sheds light on essential role players, it also demonstrates the complexities of these interactions, leaving room for more research to investigate additional influences or associations.

These findings suggest that the promotion of PA and the creation of supportive environments for children seem to have positive associations with children’s PF and MS development. Children develop motorically through engagement in PA, as increased PA provides more opportunities to promote neuromotor and MS development [6]. Improved parental awareness, regardless of employment status, is therefore essential to encourage children to engage in physical activity, primarily through physical education and health promotion programmes in schools and through community campaigns. Although parental education and income contributed substantially to MRPF, HRPF, and MS, it should be acknowledged that SES status is difficult to change. Still, practitioners can focus on areas where more intervention is needed [98] to improve unhealthy behaviour. More attention is also required to encourage girls to engage in MVPA to improve their MRPF, HRPF, and MS development.

The strength of this study is the added understanding of the unique contributions of the multiple factors that could be investigated as potential associations of the MRPF, HRPF, and MS of children between the ages of five and eight years. This is one of only a few studies conducted on such young children, thereby contributing to our understanding of the unique associations of these variables in young developing children. The study also used standardised tests to measure PF, the quality and quantity of MS, and objectively measured PA levels, thereby increasing the validity and accuracy of the results. The study’s findings, therefore, add to the existing literature. However, the study also had limitations that should be acknowledged. It used a cross-sectional study design and statistical analysis, and the results are based on a relatively small group of children. Additionally, the study’s lack of data on important contextual and behavioural factors such as school PE exposure, sleep patterns, nutritional habits, biological maturation, the built environment, and parental physical activity modelling may influence potential predictors in the association with the outcome variables (i.e., HRPF, MRPF, and MS). Future studies should consider including these variables to provide a more comprehensive understanding of the factors associated with HRPF, MRPF, and MS among children and adolescents. These findings do not represent the study’s larger population nor the whole of South Africa. This limits the generalisability of the results to larger populations. As such, it affects the generalisability of the results to other populations. Longitudinal data would have provided more insight into the cause-and-effect of these associations and is therefore recommended.

## 5. Conclusions

The findings indicate that BF%, BMI, MVPA, sex, environmental factors and SES were significantly associated with HRPF, MRPF, and MS in young children, with stronger associations for HRPH. These associations highlight their potential importance in early childhood development and the establishment of future physical activity behaviours. BF% and MVPA emerged as key associated factors, underscoring the importance of promoting active lifestyles and healthy body composition from an early age. SES (parental education, income, and employment) was also associated with several fitness and motor outcomes, underscoring the need for targeted strategies to address socio-economic disparities. Notable sex differences in MRPF and MS were observed, likely reflecting both physiological and sociocultural influences. However, given the cross-sectional design, these findings should be interpreted as associations rather than causal relationships. Overall, the results emphasise the value of supportive environments and a holistic approach to promote children’s physical fitness and motor skill development, particularly in lower-resourced contexts.

## Figures and Tables

**Table 1:** Participant age, body composition, and BMI based on body fat characteristics per gender.

	Total (n = 299)	Boys (n = 150)	Girls (n = 149)	*p*-Value of Sex Differences
	Mean ± SD	Mean ± SD	Mean ± SD	
Age (Year)	6.83 ± 0.96	6.85 ± 0.96	6.82 ± 0.97	0.75
Body height (cm)	122.58 ± 7.99	123.23 ± 7.94	121.91 ± 8.01	0.15
Body weight (kg)	24.46 ± 6.46	24.69 ± 6.57	24.24 ± 6.35	0.55
BMI (kg/m^2^)	16.08 ± 2.62	16.09 ± 2.60	16.08 ± 2.60	0.97
WC (mean)	54.89 ± 6.95	55.46 ± 6.73	54.31 ± 7.15	0.15
BF% (%)	23.45 ± 8.21	20.86 ± 7.60	26.06 ± 8.00	<0.001
Fat mass (kg; BIA)	6.73 ± 12.22	5.38 ± 3.37	6.73 ± 3.66	<0.001
FFM, BIA	18.51 ± 3.99	19.37 ± 4.19	17.64 ± 3.59	<0.001
TBW (L, BIA)	14.74 ± 5.34	15.15 ± 4.75	14.32 ± 5.86	0.18
BMI based body fatness classification				
	**Total**	**Boys** **(n = 150)**	**Girls** **(n = 149)**	**Pearson chi-square for the group differences**
	**n (%)**	**n (%)**	**n (%)**	
Underweight/Thinness FM% < 18.5%	79 (27)	57 (38)	22 (15)	<0.001
Normal FM% between 18.5 and 29.9%	162 (54)	77 (52)	85 (57)	
Overweight FM% between 30 & 34.9	33 (11)	10 (7)	23 (15)	
Obese FM% > 35%	24 (8)	5 (3)	19 (13)	

SD = standard deviation; n = number; BMI = Body mass index; WC = Waist circumference; BIA = Bioelectrical impedance; BF% = body fat percentage; FFM = Fat free mass/Lean mass; TBW = Total body water; FM = fat mass percent.

**Table 2:** MRPF and HRPF characteristics of the group and by gender.

	Motor and Fitness *r*			Boys	Girls	*p*-Value, Sex Differences
	Mean ± SD	Min	Max	Mean ± SD	Mean ± SD	
Motor-related Physical fitness (MRPF)						
Speed 10 m (sec)	2.41 ± 0.26	1.95	4.43	2.35 ± 0.21	2.47 ± 0.30	<0.001 *
Speed 20 m (sec)	4.34 ± 0. 45	2.26	6.59	4.23 ± 0.40	4.46 ± 0.48	<0.001 *
Agility (total)	42.18 ± 13.72	10.00	78.00	41.19 ± 12.68	43.18 ± 14.68	0.209
Motor skills (MS)						
Running quality (total)	7.17 ± 1.12	3.00	8.00	7.27 ± 1.09	7.07 + 0.33	0.124
Balance quantity (total)	35.50 ± 11.54	5.00	69.00	33.91 ± 11.60	37.11 ± 11.28	0.016 *
Balance quality (total)	2.34 ± 0.58	1.00	3.00	2.29 ± 0.57	2.39 ± 0.59	0.169
Catching quantity (total)	3.40 ± 1.55	0.00	5.00	3.55 ± 1.55	3.25 ± 1.55	0.090
Catching quality (total)	5.20 ± 0.96	0.00	6.00	5.23 ± 0.98	5.17 ± 0.95	0.090
Kicking quantity (total)	2.94 ± 1.33	0.00	5.00	3.20 ± 1.23	2.68 ± 1.39	<0.001 *
Kicking quality(total)	7.20 ± 1.25	1.00	8.00	7.63 ± 0.77	6.77 ± 1.48	<0.001 *
Health-related physical fitness (HRPF)						
SBJ quantity (distance)	113.08 ± 18.53	66.10	165.20	116.84 ± 18.74	109.30 ± 17.58	<0.001 *
SBJ quality (total)	6.01 ± 1.33	1.00	8.00	6.14 ± 1.28	5.87 ± 1.37	0.09
PACER (laps) (m)	27.46 ± 12.97	4.00	78.00	29.82 ± 14.65	25.10 ± 10.57	0.002 *
V^·^O_2max_ (mL/kg/min)	47.11 ± 4.30	37.85	64.40	47.95 ± 4.81	46.27 ± 3.54	<0.001 *

SD = standard deviation; SBJ = standing broad jump; *p* < 0.05 = statistical significance (*). Note: running quantity is the 10 and 20 m speed scores.

**Table 3:** PA as measured objectively by using the ActiGraph accelerometer.

	Total (n = 299)	Boys (150)	Girls (n = 149)	*p*-Value, Sex Differences
	Mean ± SD	Mean ± SD	Mean ± SD	
Sedentary behaviour (min/day)	372.33 ± 58.94	367.69 ± 62.48	399.01 ± 54.97	0.17
Light PA (min/day)	343.57 ± 54.02	347.78 ± 52.61	339.36 ± 55.27	0.18
Moderate PA (min/day)	49.79 ± 15.96	54.18 ± 17.13	45.38 ± 13.35	<0.001 *
Vigorous PA (min/day)	22.02 ± 11.68	24.10 ± 12.31	19.93 ± 10.65	0.002 *
MVPA (min/day)	71.82 ± 25.21	78.28 ± 26.89	65.31 ± 21.60	<0.001 *
PA levels using the ActiGraph accelerometer				
	n (%)	n (%)	n (%)	
Meeting Recommended daily 60 min MVPA/day	197 (66)	110 (73)	87 (58)	0.002 *
Not Meeting Recommended 60 min MVPA/day	102 (34)	40 (27)	62 (42)	

PA = physical activity; MVPA = moderate-to-vigorous physical activity; SD = standard deviation; n = number; % = percentage; *p* < 0.05 = statistical significance (*).

**Table 4:** Associated factors’ beta (Β), *p*-values, and 95% confidence intervals (CIs) for HRPF.

HRPF	*Β*	*R* ^2^	Adjusted *R*^2^	*F*	*p*	95% CI for *β*	
						Lower Bound	Upper Bound
SBJ							
BF%	−0.765	0.211	0.206	45.1417	<0.001 *	−1.051	−0.479
Education	0.161	0.266	0.257	30.551	0.037	1.153	36.360
MVPA	0.240	0.305	0.293	24.631	<0.001 *	0.082	0.290
Income	0.155	0.321	0.293	19.755	0.049	0.045	27.020
BMI	0.921	0.278	0.264	19.807	0.048	0.009	1.832
Pacer laps							
MVPA	0.309	0.136	0.131	26.547	0.003 *	0.001	0.003
BF%	−0.226	0.183	0.174	18.78	<0.001 *	−0.009	−0.002
Employment	0.878	0.131	0.121	13.010	0.007	0.788	4.968
V^·^O_2max_							
MVPA	0.351	0.151	0.146	30.052	<0.001 *	0.000	0.001
BF%	−0.119	0.145	0.138	21.916	<0.001 *	−0.180	−0.058
Employment	0.891	0.166	0.156	17.114	0.011	0.206	1.576

beta (β) = regression coefficient, *R*^2^ = coefficient of determination, adjusted *R*^2^, *p*-values and 95% CI, BF% = body fat percentage; *p* < 0.05 = statistical significance (*).

**Table 5:** Associated factors (beta (Β), *p*-values and 95% confidence interval (CI) for the beta) for MRPF and MS.

MRPF	*Β*	*R* ^2^	Adjusted R^2^	*F*	*p*	95% CI for *β*	
						Lower Bound	Upper Bound
**Speed 10 m**							
Sex	0.245	0.098	0.093	18.489	0.005 *	0.009	0.034
School quintile status	−0.171	0.156	0.156	11.526	0.010 *	−0.022	−0.005
BF%	0.201	0.138	0.127	13.483	0.007 *	0.000	0.002
**Speed 20 m**							
Sex	0.257	0.107	0.102	20.446	<0.001 *	0.011	0.035
BF%	0.009	0.118	0.111	17.358	<0.001 *	0.002	0.015
School quintile status	−0.104	0.145	0.135	14.644	<0.001 *	−0.161	−0.046
MVPA	−0.253	0.158	0.148	15.848	<0.001 *	−0.005	−0.001
**Running quality**							
BF%	−0.144	0.021	0.017	6.247	0.013 *	−0.036	−0.002
**Agility**							
MVPA	0.103	0.030	0.026	8.060	0.002 *	0.037	0.168
School quintile status	−4.836	0.047	0.059	6.474	<0.001 *	−7.428	−2.243
Income	3.092	0.094	0.089	7.393	<0.001 *	1.621	4.563
**Balance quality**							
BMI	−0.138	0.019	0.015	5.019	0.025 *	−0.526	−0.035
**Balance quantity**							
BMI	−0.223	0.050	0.045	10.821	0.001 *	−0.021	−0.002
Employment	3.409	0.036	0.028	4.825	0.002 *	1.253	5.564
School quintile status	−2.406	0.062	0.062	5.702	0.008 *	−4.169	−0.644
**Catching quantity**							
BMI	0.328	0.036	0.031	7.220	<0.001 *	0.085	0.227
MVPA	0.009	0.068	0.068	10.551	0.021 *	0.001	0.016
**Catching quality**							
BMI	0.228	0.052	0.048	11.823	<0.001 *	0.003	0.010
Employment	0.173	0.083	0.076	11.814	0.034 *	0.013	0.332
**Kicking quantity**							
Sex	−0.148	0.022	0.017	4.473	0.036 *	−0.122	−0.004
**Kicking quality**							
Sex	−0.305	0.093	0.089	21.279	<0.001 *	−0.090	−0.036

beta (β) = regression coefficient, *R*^2^ = coefficient of determination, adjusted *R*^2^, *p*-values and 95% CI, BF% = body fat percentage; * = statistical significance at *p* < 0.05.

## Data Availability

The authors confirm that the data supporting these findings are not available online but are available from the authors (Anita Pienaar) upon reasonable request, in accordance with NWU policy guidelines.

## Supplementary Materials

The following supporting information can be downloaded at https://www.mdpi.com/article/10.3390/children13050605/s1, Figure S1: Flow diagram of the three phases of the baseline study population and data collection. (**A**) The pre-screening phase including the planning, organization, permission and recruitment; (**B**) The screening phase which involved participant assent and consent, and baseline data collection; and (**C**) The post-screening phase comprises of a multifaceted feedback process and data analysis; Figure S2: Flow diagram for the larger body composition using the isotope technique (BC-IT study) study. References [65,67] are cited in supplementary materials.

## Abbreviations

The following abbreviations are used in this manuscript:

HRPF	Health-related physical fitness
MRPF	Motor-related physical fitness
PF	Physical fitness
PA	Physical activity
MS	Motor skills
CRF	Cardiorespiratory fitness
MVPA	Medium to vigorous physical activity
SES	Socio-economic status
BC	Body composition
UW	Under-weight
OW	Over-weight
OB	Obese
ISAK	International Society for the Advancement of Kinanthropometry
WHO	World Health Organisation
BIA	Bioelectrical impedance analysis
BF%	Body fat percentage
BMI	Body mass index
