# Supplementary material for: Variables Most Strongly Associated with Motor- and Health-Related Physical Fitness and Motor Skills in Five- to Eight-Year-Old Children: The BC-It and Examin Youth SA Studies
Source: Children (Basel). 2026 Apr 27;13(5):605. doi: 10.3390/children13050605 (PMC13204860; doi:10.3390/children13050605)
Supplement: Supplementary file 1 [file children-13-00605-s001.zip › children-4236602-supplementary.pdf]

Supplementary Materials [65,67]

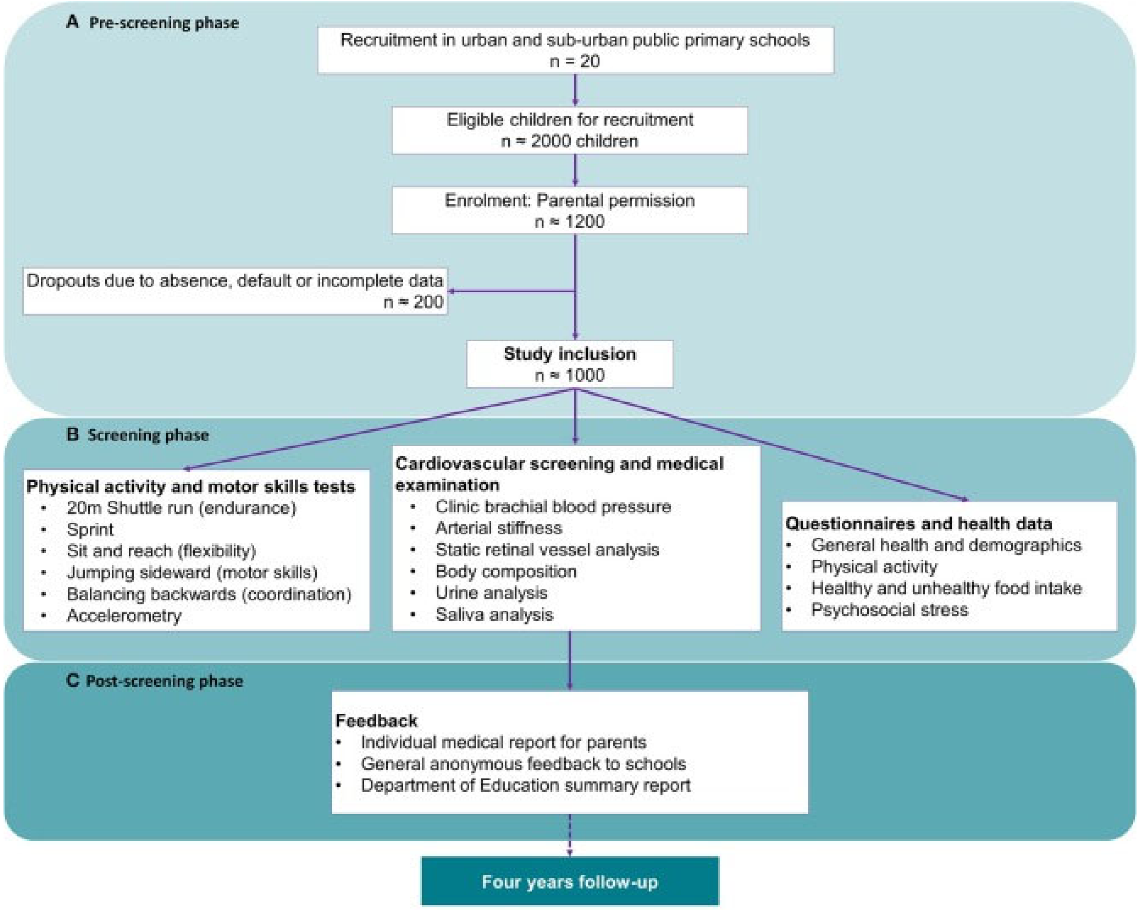

Figure S1. Flow diagram of the three phases of the baseline study population and data collection. **(A)** The pre-screening phase including the planning, organization, permission and recruitment; **(B)** The screening phase which involved participant assent and consent, and baseline data collection; and **(C)** The post-screening phase comprises of a multifaceted feedback process and data analysis.

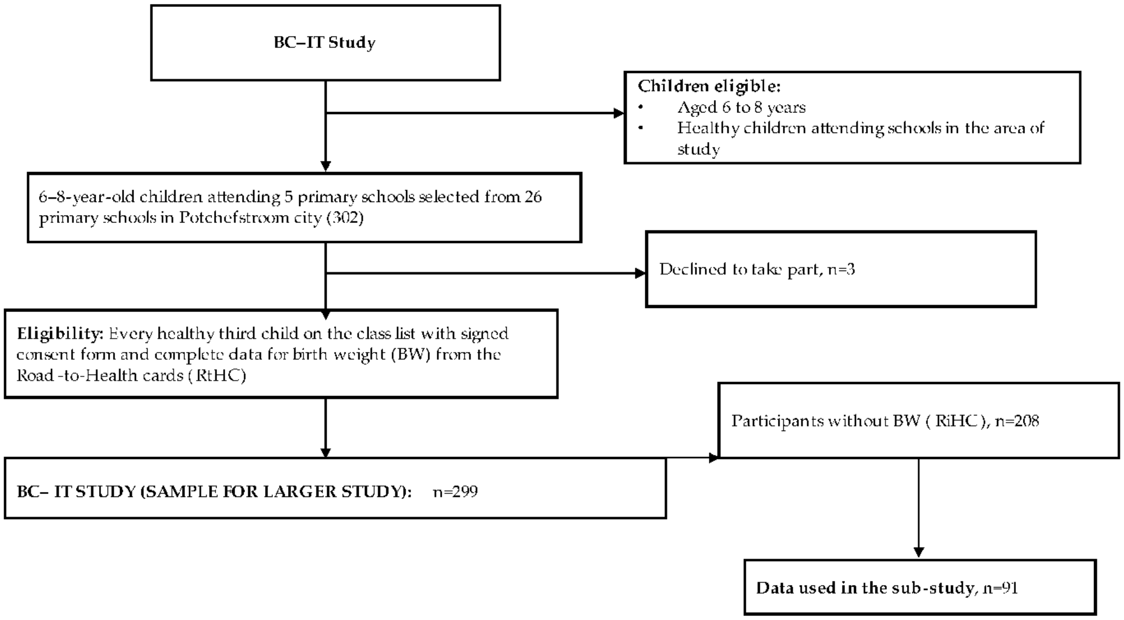

Figure S2. Flow diagram for the larger body composition using the isotope technique (BC-IT study) study.
